# Psychogenic Foreign Accent Syndrome: A New Case

**DOI:** 10.3389/fnhum.2016.00143

**Published:** 2016-04-19

**Authors:** Stefanie Keulen, Jo Verhoeven, Louis De Page, Roel Jonkers, Roelien Bastiaanse, Peter Mariën

**Affiliations:** ^1^Department of Linguistics and Literary Studies, Clinical and Experimental Neurolinguistics, Vrije Universiteit BrusselBrussels, Belgium; ^2^Department of Linguistics, Center for Language and Cognition Groningen, Rijksuniversiteit GroningenGroningen, Netherlands; ^3^Department of Language and Communication Science, School of Health, City University LondonLondon, UK; ^4^Department of Linguistics, Computational Linguistics and Psycholinguistics Research Center, Universiteit AntwerpenAntwerp, Belgium; ^5^Department of Psychology, Faculty of Psychology and Educational Sciences, Vrije Universiteit BrusselBrussels, Belgium; ^6^Department of Neurology and Memory Clinic, ZNA Middelheim General HospitalAntwerp, Belgium

**Keywords:** foreign accent syndrome, psychogenic FAS, speech disorder, psychodiagnostics, accent attribution experiment, accent rating experiment

## Abstract

This paper presents the case of a 33-year-old, right-handed, French-speaking Belgian lady who was involved in a car accident as a pedestrian. Six months after the incident she developed a German/Flemish-like accent. The patient's medical history, the onset of the FAS and the possible psychological causes of the accent change are analyzed. Relevant neuropsychological, neurolinguistic, and psychodiagnostic test results are presented and discussed. The psychodiagnostic interview and testing will receive special attention, because these have been underreported in previous FAS case reports. Furthermore, an accent rating experiment was carried out in order to assess the foreign quality of the patient's speech. Pre- and post-morbid spontaneous speech samples were analyzed phonetically to identify the pronunciation characteristics associated with this type of FAS. Several findings were considered essential in the diagnosis of psychogenic FAS: the psychological assessments as well as the clinical interview confirmed the presence of psychological problems, while neurological damage was excluded by means of repeated neuroimaging and neurological examinations. The type and nature of the speech symptoms and the accent fluctuations associated with the patient's psychological state cannot be explained by a neurological disorder. Moreover, the indifference of the patient toward her condition may also suggest a psychogenic etiology, as the opposite is usually observed in neurogenic FAS patients.

## Introduction

Foreign accent syndrome (FAS) is a rare motor speech disorder which causes patients to speak their native language with an accent which is perceived as non-native by speakers of the same speech community. This “non-nativeness” is the result of suprasegmental and/or segmental changes, which—according to the criteria proposed by Whitaker (1982)—are the consequence of damage to the central nervous system. Often, the etiology is stroke or brain trauma affecting the language dominant areas of the brain, e.g., the left (pre)frontal, temporal and/or parietal region, the rolandic and perisylvian area, as well as the insular region. Nevertheless, FAS has also been associated with other etiologies including MS (Villaverde-Gonzalález et al., 2003; Bakker et al., 2004; Chanson et al., 2009), neoplasms (Abel et al., 2009; Masao et al., 2011; Tomasino et al., 2013) and vascular dementia (Paquier and Assal, 2007). Verhoeven and Mariën (2010) argue that FAS is not only caused by (acute) neurological damage but it can also result from psychogenic issues. In psychogenic FAS, the accent is associated with a psychological/psychiatric disorder. Furthermore, Verhoeven and Mariën (2010) also identified a mixed type in which FAS initially develops on the basis of a neurological disorder: this affects patients so profoundly that they further develop the accent in order to create the impression of a more authentic personality.

The current study focuses on psychogenic FAS. For most of the psychogenic cases reported so far, a psychogenic cause was assumed because it was not possible to unambiguously identify a neurological disorder. Some authors have discarded the idea of psychogenic FAS because of diagnostic difficulties to objectify this condition (Gurd et al., 2001; Poulin et al., 2007). In some patients diagnosed with psychogenic FAS (repeated) brain imaging with CT or MRI revealed structural damage, but the speech problems were disproportionate in relation to the damage. Furthermore, in the majority of the psychogenic FAS cases symptoms were fluctuating, increasing in certain (social/emotional) contexts, diminishing or even completely resolving in others (e.g., Van Borsel et al., 2005; Tsuruga et al., 2008; Haley et al., 2010; Jones et al., 2011). Such a atypical fluctuating course of symptoms is typical of speech and voice disorders of psychogenic origin (Avbersek and Sisodiya, 2010). When FAS is typified by these phenomena and associated with identifiable psychological problems (e.g., depression, familial history, suicidal ideation) a non-organic origin may be expected (Roth et al., 1989; Tippett and Siebens, 1991; Baumgartner and Duffy, 1997; Baumgartner, 1999).

## Background

In little over a century—counting from the first (anecdotal) FAS description by Pierre Marie in 1907 until July of 2014—only 15 FAS cases with a presumed psychogenic origin have been reported (Critchley, 1962; Gurd et al., 2001; Reeves and Norton, 2001; Van Borsel et al., 2005; Verhoeven et al., 2005; Poulin et al., 2007; Roy et al., 2012, case 1; Reeves et al., 2007; Tsuruga et al., 2008; Cottingham and Boone, 2010; Haley et al., 2010; Jones et al., 2011; Lewis et al., 2013; Polak et al., 2013). This study presents a new case of psychogenic FAS. Neuropsychological testing was carried out to assess a wide range of cognitive functions. The psychological state of the patient was evaluated by means of a series of psychodiagnostic tests, including symptom validity tests. Extensive neuropsychological investigations (Verhoeven et al., 2005; Poulin et al., 2007; Haley et al., 2010) and psychodiagnostic testing (Verhoeven et al., 2005; Cottingham and Boone, 2010) have only been occasionally reported in psychogenic case reports, although such an in-depth investigation is crucially important for accurate diagnosis and successful therapy (see also: Moreno-Torres et al., 2013). In addition, a perceptual analysis of the patient's most salient speech characteristics was carried out and an accent rating experiment was run to find out to what extent the patient's accent was considered as non-native. Additionally, the listening panel was asked to indicate the mother tongue of the FAS speaker. Such experiments have previously only been reported in four other studies (Di Dio et al., 2006; Kanjee et al., 2010; Verhoeven et al., 2013: rating and attribution experiment; Dankovičová and Hunt, 2011: rating experiment). We are convinced that perceptual assessment reinforces the diagnosis of FAS and it may provide new insights into the perceptual impression(s) created by FAS in the ear of the beholder (Verhoeven et al., 2013).

The patient gave written informed consent to report the medical data. All the tests reported below are part of the standard, clinical neurolinguistic work-up in patients with speech and language disorders at ZNA Middelheim general hospital. Speech recordings were also made to allow for better follow-up. The patient gave written consent to use recorded speech samples for the perceptual evaluation in a public environment.

### Case presentation and medical history

SB is a 33-year-old, right-handed, monolingual French-speaking lady, originating from a village in the francophone Walloon part of Belgium near the Flemish border. She was raised in French and her parents were monolingual French-speaking Belgians. From a neurological perspective, growth and development were unremarkable. There was no family history of neurodevelopmental disorders or learning disabilities. She had always obtained normal school results and had an educational level of 12 years. She consulted the neurology department in November 2013 because of a “Dutch or German-like accent,” which she acutely developed approximately 6 months after she was hit by a car while crossing the street to deliver orders from the bakery where she worked as a saleswoman. A few months after the accident occurred, the patient mentioned an “abrupt change of personality.” She considered her behavioral change as the cause for her sudden dismissal at work. There had been serious disagreements with colleagues, customers, as well as with her line manager. She was dismissed in June 2012. It was shortly after her dismissal that she developed a foreign accent.

The accident happened in December 2011. There had been no loss of consciousness. Apart from some superficial subcutaneous hematomas in the frontal and right peri-orbital region, clinical examination on admission to the hospital was normal. CT scan of the brain and spinal cord were normal. A diagnosis of minor head trauma was made. One week later, the patient started suffering from increasingly painful headaches (possibly a post-traumatic migraine, see: Weiss et al., 1991) and a desensitization of the scalp. She complained of vertigo and was hospitalized for 3 days. The clinical neurological examination on admission was normal. Laboratory investigations (blood and urine), EEG and CT were normal as well. She was diagnosed with a post-concussion syndrome, benign paroxysmal vertigo (positive Hallpike test) and a cervical trauma. Approximately 1 month later the symptoms were still present. She identified several regions of hyperaesthesia and anesthesia in the facial area and the scalp. The vertigo had receded, but she complained of severe neck and shoulder pain. Approximately 4 months after the accident, she consulted a neurologist again. The clinical neurological examination and EEG revealed no abnormalities. During this visit, the patient mentioned that she felt she had become “someone else” after the accident, with regular aggressive outbursts toward family, friends, strangers, and clients. The patient complained about attention deficits and permanent fatigue. She also mentioned that the intensity of the accent was fluctuating: the accent was heavier when she was tired.

Due to the persistence of her complaints with respect to her accented speech and memory, the patient was referred to hospital for additional radiological examinations. In November 2012, she underwent a saggital T1-weighted and axial FLAIR, diffusion, SWI, proton density and T2-weighted MRI of the head, a coronal FLAIR MRI perpendicular to the axes of the left and right hippocampi, as well as an angio-MRI of the brain and 3D TOF of the circle of Willis. The qualified radiologist reported that all acquisitions were normal.

In November 2013, she consulted our department because of the persistence of the accent change and cognitive complaints (attention problems and episodes of confusion). At a linguistic level she suffered from word-finding difficulties and morphological problems related to article-noun agreement (she did not differentiate between the masculine and feminine forms of the definite article). According to her, listeners had the impression that she spoke with a Dutch accent. Her previous customers, for instance, had perceived her as a native Dutch-speaking Belgian and repeatedly asked her why she spoke French instead of “Flemish” (the Belgian variant of Dutch; see: Verhoeven, 2005). She still suffered from behavioral changes and avoided social contact with her family and friends because of a lack of interest on her part. Yet, she was looking for more excitement in life, as well as a more frivolous, out-going lifestyle. She said she was deeply bored. In addition, a number of depressive symptoms were mentioned including apathy, loss of drive and initiative, and mood-swings.

### Neuropsychological testing

The first neuropsychological assessments were carried out approximately 1 year after the accident in January 2012 (see Table 1 for an overview of the results). The test battery consisted of the Wechsler Adult Intelligence Scale-IV (WAIS-IV; Wechsler, 2011, French Ed.), the d2-test (Brickenkamp and Zillmer, 1998), the “Barrage de Zazzo” (Zazzo, 1974), the Stroop Test (Stroop, 1935), the Wisconsin Card Sorting Test (WCST; Grant and Berg, 1948), and the California Verbal Learning Task (CVLT, Delis et al., 2000). Repeated neuropsychological testing in 2014 consisted of the Wechsler Memory Scale—Revised (Wechsler, 1987), the Boston Naming Test (Kaplan et al., 1983); the Trail Making Test (Reitan, 1958), and the d2-test.

**Table 1:** **Neuropsychological test results for the years 2012 and 2014**.

**Neuropsychology**					
**Tests 2012**	**Scaled scores (raw score)**	**Mean (± 1SD) *Z-score (pct.)***	**Tests 2014**	**Scaled scores (raw score)**	**Mean (± 1SD) *Z-score (pct.)***
Intelligence					
WAIS-IV					
FSIQ	105	100 (±15)			
Verbal comprehension scale	96	100 (±15)			
- similarities	(10)	10 (±3)			
- vocabulary	(9)	10 (±3)			
- information	(9)	10 (±3)			
Working memory scale	112	100 (±15)			
- arithmetic	(14)	10 (±3)			
- digit span	(10)	10 (±3)			
Perceptual organization scale	120	100 (±15)			
- block design	(13)	10 (±3)			
- matrix reasoning	(14)	10 (±3)			
- picture completion	(13)	10 (±3)			
Processing speed scale	86	100 (±15)			
- symbols	(7)	10 (±3)			
- coding	(8)	10 (±3)			
Attention d2-test			Attention d2-test		
- Total items (Tn)	(249)	*Z* = −3.08	- Total items (Tn)	(242)	*Z* = −2.44
- Total (corrected for mistakes) (Tn-F)	(246)	*Z* = −2.94	- Total (corrected for mistakes) (Tn-F) - Concentration (C-F2)	(242) (105)	*Z* = −2.20 *Z* = −1.68
- Variation in tempo (Tn highest-Tn lowest)	(7)	*Z* = −1.15	- Variation in tempo (Tn highest-Tn lowest)	(10)	*Z* = 0.5
Barrage de Zazzo (10 min.)					
Fastness	(103.6)	*(pct. 12.5–25)*			
Exactness	(11.48)	*(pct. 25–50)*			
Profitableness	(239)	*(pct. 25–37.5)*			
Executive functions Wisconsin Card Sorting Test			Executive functions Trail Making Test		
Nr. of categories realized	6		- Test A	(52″ 38)	*(pct. < 10)*
Learning capacity	24.25%		- Test B	(1′38″ 25)	*(pct. 20)*
Nr. of errors	7 (69)				
Stroop					
Naming	(89″)	64.78 (±16.25)			
- Mistakes	2	1.13 (±1.59)			
Reading	(39″)	46.72 (±16.4)			
- Mistakes	1	0.38 (±0.72)			
Interference	(155.8″)	111 (±27.58)			
- Mistakes	3	3.5 (±4.15)			
Flexibility	(221″)	133.52 (±52)			
- Mistakes	6	2.89 (±2.61)			
(Long Term) Memory California Verbal Learning Test			Memory		
List A	(66)	57.88 (±5.46)	WMS-R		
Total 1–5	(5)	7 (±2.37)	Attention/Concentration	(50) 70	100 (±15)
List B			- mental control	(4)	10 (±3)
Free recall of A	(14)	12.35 (±1.97)	- number series	(18)	10 (±3)
Cued recall A	(16)	13 (±1.90)	- visual series	(28)	10 (±3)
Delayed recall A	(15)	13 (±1.84)	Visual Memory	(66) 133	100 (±15)
Cued delayed recall A	(16)	13.59 (±1.91)	- perceptual memory	(7)	10 (±3)
Recognition	(16)	14.71 (±1.40)	- associated visual pairs	(18)	10 (±3)
			- visual reproduction	(41)	10 (±3)
			Verbal Memory	(42) 74	100 (±15)
			- logical memory	(26)	10 (±3)
			- associated verbal pairs	(16)	10 (±3)
			Global Memory	(108) 86	100(±15)
			Delayed Recall	(74) 91	100 (±15)
			- logical memory	(11)	10 (±3)
			- associated visual pairs	(12)	10 (±3)
			- associated verbal pairs	(14)	10 (±3)
			- visual reproduction	(16)	10 (±3)
			Language Boston Naming Test (/60)	(53)	

FSIQ, full scale IQ; WAIS-IV, Wechsler Adult Intelligence Scale—IV; WMS-R, Wechsler Memory Scale; Stroop, Stroop task.

A full scale IQ (FSIQ) of 105 was found with a significant discrepancy of 24 IQ-points between the verbal (96) and performance IQ level (120). All subtest scores were within the normal range. Executive function (mental flexibility, frontal problem solving) was tested by means of the Stroop and the WCST. She obtained a normal result on the WCST, but depressed scores on the Stroop with slowed processing in the color naming condition (Z-score = −1.5 SD), interference condition (Z-score = −1,6 SD), and flexibility condition (Z-score = −1.7 SD). Tests measuring sustained visuo-motor and selective attention (d2-test in 2012/2014 and the “test de barrage de Zazzo”) were performed at a slow pace. Scores for total items treated for the d2-test (2012: Z = −3.08 SD; 2014: Z = −2.44 SD) as well as the total items corrected (2012: Z = −2.94 SD; 2014: Z = −2.20 SD) were in the pathological range. As shown by the CVLT, verbal memory was intact, the patient obtained borderline results for the “total recollection” (5 trials) of List A (Z-score = −1.49 SD). On other subtasks of the CVLT she obtained normal results (+1 SD: Cued recall A, Delayed recall A, Cued delayed recall A, Recognition).

In 2014, a significant discrepancy between a very superior visual memory index (= 133) and clinically deficient verbal memory index (= 74; −1.7 SD) was found on the WMS-R. As reflected by a general attention index of 70 (−2 SD), the WMS-R tasks scores were in the deficient range. The Trail Making Test (part A and B) disclosed low average visual search (< pct. 10) and mental flexibility (pct. 20). Sustained visuo-motor attention scores were within the defective range. Performance on the BNT was normal. Overall, the data for the test session in 2014 were in line with the results obtained in 2012.

### Psychodiagnostic assessment

The psychodiagnostic assessment consisted of an interview with an experienced clinical psychologist (LDP), which was followed some time later by a session during which the patient was asked to respond to a series of standardized questionnaires. These questionnaires were completed at the hospital, without the help of the examiner. Testing included the Minnesota Multiphasic Personality Inventory-2 (MMPI-2: Butcher et al., 1989); the Defense Style Questionnaire (DSQ-60: Thygesen et al., 2008); the Rotter Incomplete Sentences Blank (RISB-FR: Rotter et al., 1992); Beck Depression Inventory-II (BDI-2: Beck et al., 1996), Pathological Narcissism Inventory (PNI: Pincus et al., 2009; French version: Diguer et al., 2014), and the Narcissistic Personality Inventory-40 (NPI-40: Raskin and Hall, 1979).

Furthermore, symptom validity and self-presentation tests were carried out by means of the List of Indiscriminate Psychopathology (LIPP: Merten and Stevens, 2012), and the Supernormality Scale (SS: Cima et al., 2003). The LIPP is an experimental questionnaire, which measures calibration problems. It consists of questions addressing pseudo- and real symptoms (Merckelbach et al., 2013). Malingering participants are in doubt as to which symptoms they can report and which ones they cannot. The SS is a questionnaire, which evaluates deception or denial under the guise of giving socially desirable answers (Cima et al., 2003).

During intake the patient gave evidence of disinhibition which mainly manifested itself as laughing without reason, Witzelsücht and inappropriate comments. The patient was reticent and maintained a (psychologically immature) defensive attitude throughout the entire interview. Her thoughts were preoccupied by frustration about her own situation. The interview was dominated by her feelings concerning her increased impulsiveness, aggressiveness and apathetic demeanor vis-à-vis her family, former boss, and colleagues. The examiner noticed that a topic which rendered her frustrated led to an emotional breakthrough during which she lost the “Dutch/Flemish-like” accent. The patient's interview contained numerous contradictions (e.g., stating at first that she was a very lively, out-going person, but when asked later what she did during the day, she answered that she sat in a chair as all personal contact bored her and conversations with others—even friends—were too difficult and tiring). The description of her emotional and family life remained superficial and prosaic. The interview revealed increasing relational problems. The relationship with her husband left her “unaffected” and relationships with friends, family and relatives were unstable, marked by serious rows in which she responded unpredictably.

She confirmed egocentric and narcissistic tendencies. It was not possible to detect signs of perceptual aberration or other florid psychotic symptoms. A few weeks after the interview, a series of standardized psychodiagnostic tests were administered. Symptom validity and self-presentation tests, such as the List of Indiscriminate Psychopathology and the Supernormality Scale, did not yield indications for (conscious or unconscious) manipulation. Personality testing indicated a wide, undifferentiated personality disturbance. Interestingly, scores on both narcissism measures (NPI and PNI) were at most extreme upper ends, which is consistent with her answers during the clinical interview. A thymic disturbance and affective lability were objectified (APA, 2000; DSM-IV-TR, Axis I), but test results did not equivocally point toward a well-defined personality disturbance. Clinically, however, the patient gave clear indications of highly dependent, histrionic and borderline personality characteristics (APA, 2000; DSM-IV-TR, Axis II). On a psychodynamic structural level, she was considered to have a borderline personality organization level of functioning (Kernberg, 1984), because of an immature defensive functioning, intact reality testing, but severe lack of personality integration. This is relevant in relation to (interpersonal) acting out and poor bodily representation. The overall clinical presentation seemed chronic, pervasive and well established throughout her psychic development.

### Perceptual analysis of spontaneous speech sample

A post-morbid speech sample was recorded in November 2013. It consisted of 5 min of video-recorded spontaneous speech, which was selected from an interview with the patient. In this interview she talks about her accent change and her relational and professional problems. This sample consisted of 644 words (including filled pauses). The patient also provided two (short) pre-morbid speech samples consisting of 43 and 26 s of conversational speech dating from April and July 2011, i.e., approximately half a year before the accident. When comparing pre- and post-morbid speech samples a number of striking differences were found. The first one was a very strong trilling aspect when realizing the uvular [R]. The trill is too excessive for French, and is more typical of the one in German and some regional variants of Dutch (36/644). According to Van de Velde and Van Hout, (1999, p. 178) “realizations of /r/ in standard Dutch until recently were the trilled realizations [R] and [r], with the uvular trill gaining in frequency and prestige especially in the Netherlands (Van Haeringen, 1924; Zwaardemaker and Eijkman, 1928; Blancquaert, 1934; Hol, 1951; Damsteegt, 1969; Mees and Collins, 1982; Vieregge and Broeders, 1993), but recently also in Flanders (Rogier, 1994).” For German this variant has been described as the most common allophone (Hall, 1993): the uvular trill-R constitutes a free (dialectal) variant of /r/, existing alongside the approximant /r/ (see Hall, 1993; Schiller, 1998). The excess trilling is particularly common in a prevocalic position (*r*aconter, *r*enverse, t*r*aite, …: 27x), less frequent in intervocalic position (di*r*ect: 1x) and postvocalic position (renve*r*se, qua*r*t, …:8x).

On the suprasegmental level, speech rate and articulation rate were particularly slow (speech rate: 2.67 syll/s, articulation rate: 3.813 syll/s). Avanzi et al. (2012) found a mean speech rate of 4.7 syll/s (SD: 0.7) and an articulation rate of 5.6 syll/s (SD: 0.6) for Belgian French of the Tournai region; the region our patient originated from. Melody and intonation appeared normal. In order to analyze rhythm, the Pairwise Variability Index was calculated (Low et al., 2001). Vocalic PVI amounted to 54.3. This is considerably higher than the accepted value for French (43.5), and is more in the range of the stress-timed languages, such as English (57.2), or German (59.7). However, the value is substantially lower than 65.5, which is the reference value for Dutch (Grabe and Low, 2002). It is also worth mentioning that the patient did not realize any liaisons, a phenomenon by which a latent word-final consonant preceding a word starting with a vowel becomes audible. Our patient failed to realize this connection for “c'est arrivé” and “tout est important”. Moreover, she did not realize the elision^1^ in “j'entends” (pronounced as “je^*^ *entends”*).

Grammar was perceived to be more simplistic than would be expected from a native-speaker of French. Sentences were perceived to be very short. At the morphosyntactic level the patient omitted the article “le” (1/644) as well as “de” in “là *de*dans,” which was realized as “là dans” (2/644). In addition, the patient made six morphological errors against the definite article. In 5 instances, the patient used the masculine definite article instead of the female form (la même chose → *le* même chose; la tête → *le* tête; ma maison → *mon* maison; la pire chose → *le* pire chose; la chose → *le* chose).

## Perceptual assessment of the foreign accent

### Aims

The foreign accent of the patient was assessed by a listening panel who listened to speech stimuli of the patient that were mixed with those of a native speaker of French and three non-native speakers with a clear foreign accent. The listening panel was required to rate the degree of foreignness and they were asked to identify the mother tongue of each of the speakers. The ratings provide additional support for the diagnosis of FAS, whereas the accent attribution gives an indication of whether naive listeners are able to perceptually identify the mother tongue of native (including the FAS patient) and non-native speakers of French. Furthermore, there was an interest to investigate whether there would be any differences between the FAS patient, the true non-native speakers and the native speaker of French.

### Methods

#### Materials and samples

Thirty students of French linguistics were recruited at the Université Libre de Bruxelles (ULB) in Brussels (age: 16–24, mean age: 20 years, 12 male and 18 female) and they were asked to rate the degree of “foreign-ness” of five speakers and to determine their native language. The students had no formal experience with speech and language pathology.

The stimuli for this experiment were taken from the intake interview, in which the patient explains what had happened to her (accident), and elaborates on her relational and professional problems. From this interview, 6 words, 3 phrases, and 6 sentences were chosen (see also: Dankovičová and Hunt, 2011). Care was taken that (a) the medical status of the FAS patient could not be derived from the stimuli and (b) the stimuli did not contain any morphological mistakes (as this could possibly influence the ratings of the listener panel). Stimulus selection was carried out by means of PRAAT, version 5.4 (PRAAT for Mac; Boersma and Weeninck, 2014).

#### Speakers

The speakers in this experiment were the FAS patient and four control speakers (Table 2) who were matched for gender with the FAS patient. The mean age of the controls was 35 years and 10 months, with an age range from 27 to 48 years old. Two speakers were Belgian but one was French-speaking and the other was Dutch-speaking (or “Flemish”; see also: Verhoeven, 2005). A third control subject spoke both Dutch and (American) English, as she was born in the USA, but moved to the Netherlands 1 year later. She was raised in English, but her education as of the age of 3 had been entirely in Dutch (100% immersion; early bilingual; see also: Bhatia and Ritchie, 2013). She no longer had contact with relatives in the USA and lived alone in the Netherlands. She considered Dutch to be her dominant language. The fourth speaker was a Russian female. No attempt was made to match the accents to those that had been informally reported for the FAS patient. It was regarded likely that most listeners were familiar with the foreign accents of the control speakers. The control speakers read the 15 stimuli that had been selected from the speech of the FAS patient. The stimuli were recorded by means of a Marantz Professional PMD 661 portable recorder and manipulated for the purpose of this experiment via PRAAT (version 5.4, 2014).

**Table 2:** **Overview of the demographic characteristics of the FAS patient and the healthy, matched controls, including an indication of the level of French (CEFR, Common European Framework of Reference for Languages)**.

**Nature**	**Gender**	**Age**	**Country of birth**	**Mother tongue**	**Level in French (CEFR)**
FAS	F	33	Belgium	French	_
Control 1	F	36	Belgium	French	_
Control 2	F	48	Belgium	Dutch (Flemish)	B2
Control 3	F	27	United States of America^*^	English/Dutch (Netherlands)	B2
Control 4	F	35	Russia	Russian	A2+/B1

* *Control 3 moved to the Netherlands one year after she was born. She was raised in English and learned Dutch as of the age of 3. Her education (immersion, 100%; early bilingual) has been entirely in Dutch*.

#### Stimuli and assessment

The perception experiment contained a total of 75 stimuli, i.e., 15 stimuli × 5 speakers. Each presentation block consisted of one stimulus read by the five different speakers. The order of the speakers differed for each block (in pseudo-random order). The stimuli were separated by a 15 s. pause to provide time for listeners to record their judgments. Total duration was 26 min. 26 s. The stimuli were played to the listeners in open field at their institution. The instructions to the test were given orally to the listening panel, but they were also able to read them. Raters provided demographic information (age, gender, country of origin, time in Belgium—if not born here, mother tongue, and other spoken languages including an indication of proficiency) in a short questionnaire. For the experiment, they were asked to first rate the “foreign-ness” of the speaker on a scale from 1 to 7. This scale is to be interpreted as a continuum ranging from “definitely *not* a native speaker of French” (= 1) to “definitely a native speaker of French” (= 7). If their response was anything other than 7, they were asked to indicate the mother tongue of the speaker (second part).

### Results

#### Statistical analysis of the accent rating experiment

The data were processed statistically in SPSS version 22 (IBM Corp., 2013). First, inter-rater reliability was tested for each speaker by calculating the intraclass correlation coefficient (ICC). A two-way random model was chosen, as each item was assessed by each of the 30 raters and raters represented a randomly selected sample. Data were checked for agreement implying that systematic differences between raters were taken into account. For FAS: ICC_(2, 30)_ = 0.94, French: ICC_(2, 30)_ = 0.903, Dutch(Be): ICC_(2, 30)_ = 0.955, English/Dutch(Nl): ICC_(2, 30)_ = 0.959, and for Russian: ICC_(2, 30)_ = 0.523.

Table 3 provides a summary of the descriptive statistics including means, standard deviations, minima and maxima, range as well as interquartile range for each of the five speakers. Based on the means (*x*) as well as median (M) it is clear that the FAS speaker is situated roughly in the middle of the seven-point scale (*x* = 3.791; σ = 2.318 and *M* = 4). The standard deviation was high, which indicates that the raters may have experienced some difficulty identifying the accent.

**Table 3:** **Overview of mean, median, standard deviations, minimum, maximum, range and interquartile range for the scores attributed to each speaker on a seven-point scale: 1, Definitely not a native speaker of French; 7, Definitely a native speaker of French**.

**Speaker**	**Mean**	**Median**	**Standard deviation**	**Minimum**	**Maximum**	**Range**	**Interquartile range**
FAS	3.791	4.000	2.318	1.000	7.000	6.000	5.000
French	6.098	7.000	1.675	1.000	7.000	6.000	1.000
Dutch (Be)	3.138	3.000	2.161	1.000	7.000	6.000	4.000
English/Dutch (Nl)	3.011	2.000	2.219	1.000	7.000	6.000	4.000
Russian	1.407	1.000	0.913	1.000	7.000	6.000	0.000

Application of the Kolmogorov-Smirnov test indicated that the data were not normally distributed (Kolmogorv-Smirnov: *p* < 0.1). Hence, non-parametric testing was applied. A Kruskall-Wallis *H* test was carried out to test whether there was a significant difference between the scores attributed to the different speakers. Results for the Kruskall-Wallis *H* test indicated that this was the case: *H*_(5)_ = 1393.60, *p* < 0.0001. However, additional Mann-Whitney *U* tests (see Table 4) were carried out to identify the speakers who differed significantly from each other and who did not. All speaker differences were significant (*p* < 0.0001), except for one: Dutch (Be) and English/Dutch(Nl) (*p* > 0.003: Bonferroni correction; *p* = 0.290).

**Table 4:** **Overview of the inter-speaker comparisons (Mann-Whitney *U*-tests)**.

**Speaker comparison**	**N stimuli**	**Mean ranks**	**Sum of mean ranks**	**Mann Whitney U**	**Wilcoxon W**	***Z***	***p***
FAS	450	322.88	145294.50	43819.500	145294.500	−15.497	0.000
French	450	578.12	260155.50				
FAS	450	487.20	219240.00	84735.00	186210.00	−4.326	0.000
Dutch(Be)	450	413.80	186210.00				
FAS	450	494.07	222331.50	81643.500	183118.500	−5.154	0.000
English/Dutch(Nl)	450	406.93	183118.50				
FAS	450	587.61	264424.50	39550.500	141025.500	−17.102	0.000
Russian	450	313.39	141025.50				
French	450	606.39	272845.00	31100.00	132575.000	−18.728	0.000
Dutch(Be)	450	294.61	132575.00				
French	450 450	604.38	271972.00	32003.000	133478.000	−18.576	0.000
English/Dutch(Nl)	450	296.62	133478.00				
French	450	659.11	296598.00	7377.000	108852.000	−25.523	0.000
Russian	450	241.89	108852.00				
Dutch(Be)	450	459.37	206717.50	97257.500	198732.500	−1.059	0.290
English/Dutch(Nl)	450	441.63	198732.50				
Dutch(Be)	450	558.65	251391.50	52583.5	154058.500	−13.864	0.000
Russian	450	342.35	154058.50				
English/Dutch(Nl)	450	547.76	246494.00	57481.000	158956.00	−12.628	0.000
Russian	450	353.24	158956.00				

**Figure 1:**
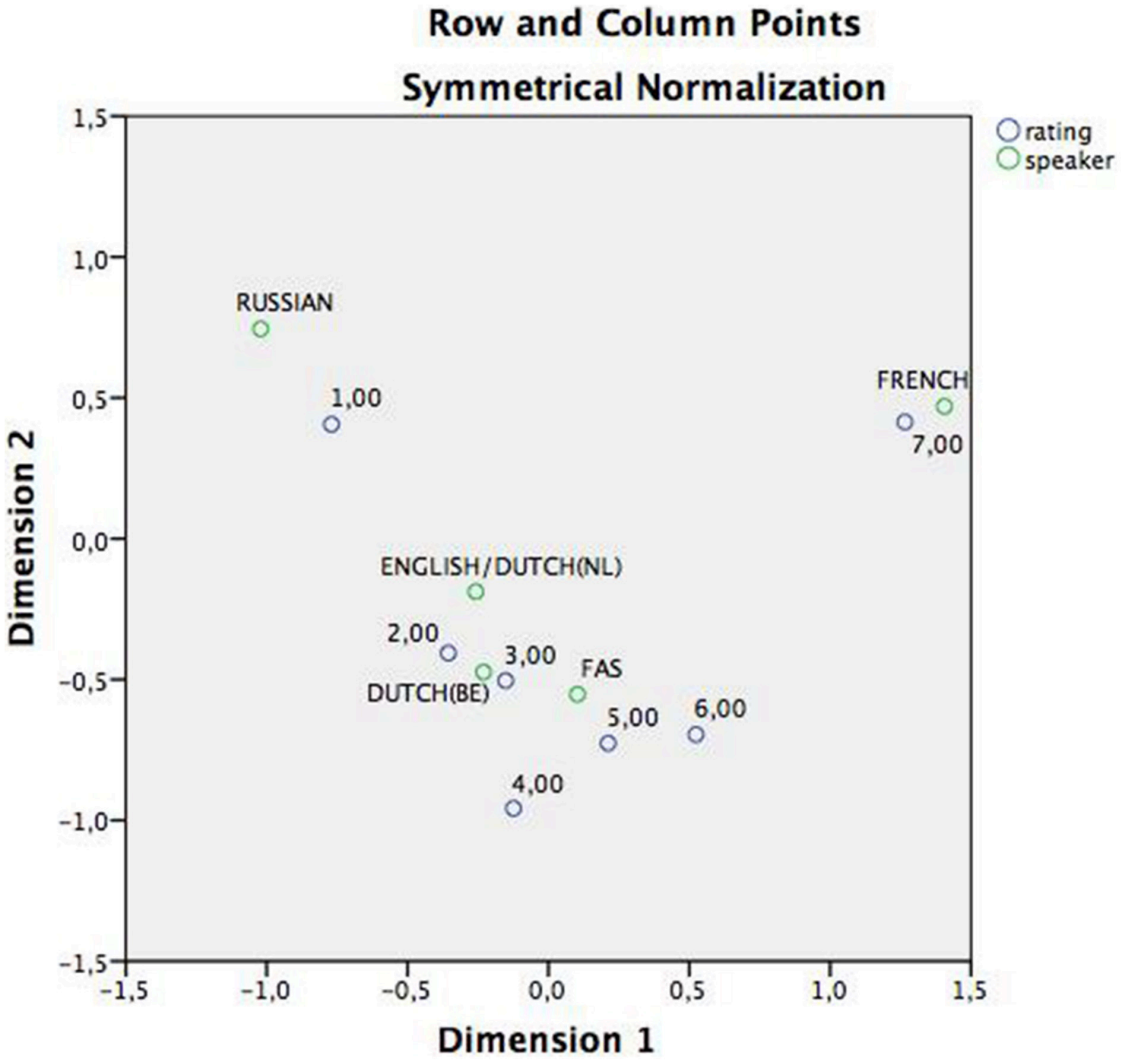
**Correspondence analysis, displaying the associations between speakers and rating in a two-dimensional plan**. As can be derived from the figure, both the Russian and the French speaker maintain an isolated position in the plain and are associated with opposite extremes of the continuum. The English/Dutch(Nl), Dutch(Be) and FAS speaker on the hand, are all grouped around the center ratings: 2,3,4, and 5.

A correspondence analysis was performed to get a two dimensional image of the strength (distance) of the associations between rating and speakers, based on frequency counts (Table 5: correspondence table; Figure 1). This showed that the associations between the native French speaker and rating “7” were particularly strong. The FAS speaker was situated more toward the higher ratings (4, 5, 6, 7) than, for instance, both native Dutch speakers and even markedly more so than the Russian speaker (strongly associated with rating “1”), who clearly occupied a more isolated position on the two-dimensional plot.

**Table 5:** **Correspondence table with frequency data for the different speakers**.

**Correspondence table**								
**Speaker**	**Rating**							
	**1**	**2**	**3**	**4**	**5**	**6**	**7**	**Active margin**
FAS	118	62	40	42	45	50	93	450
French	18	15	21	10	34	41	311	450
Dutch(BE)	165	59	51	38	51	33	53	450
English/Dutch(NL)	183	62	47	35	36	22	65	450
Russian	349	53	27	11	8	1	1	450
Active margin	833	251	186	136	174	147	523	2250

1, Definitely not a native speaker of French; 7, Definitely a native speaker of French.

#### Mother tongue identification

**Figure 2:**
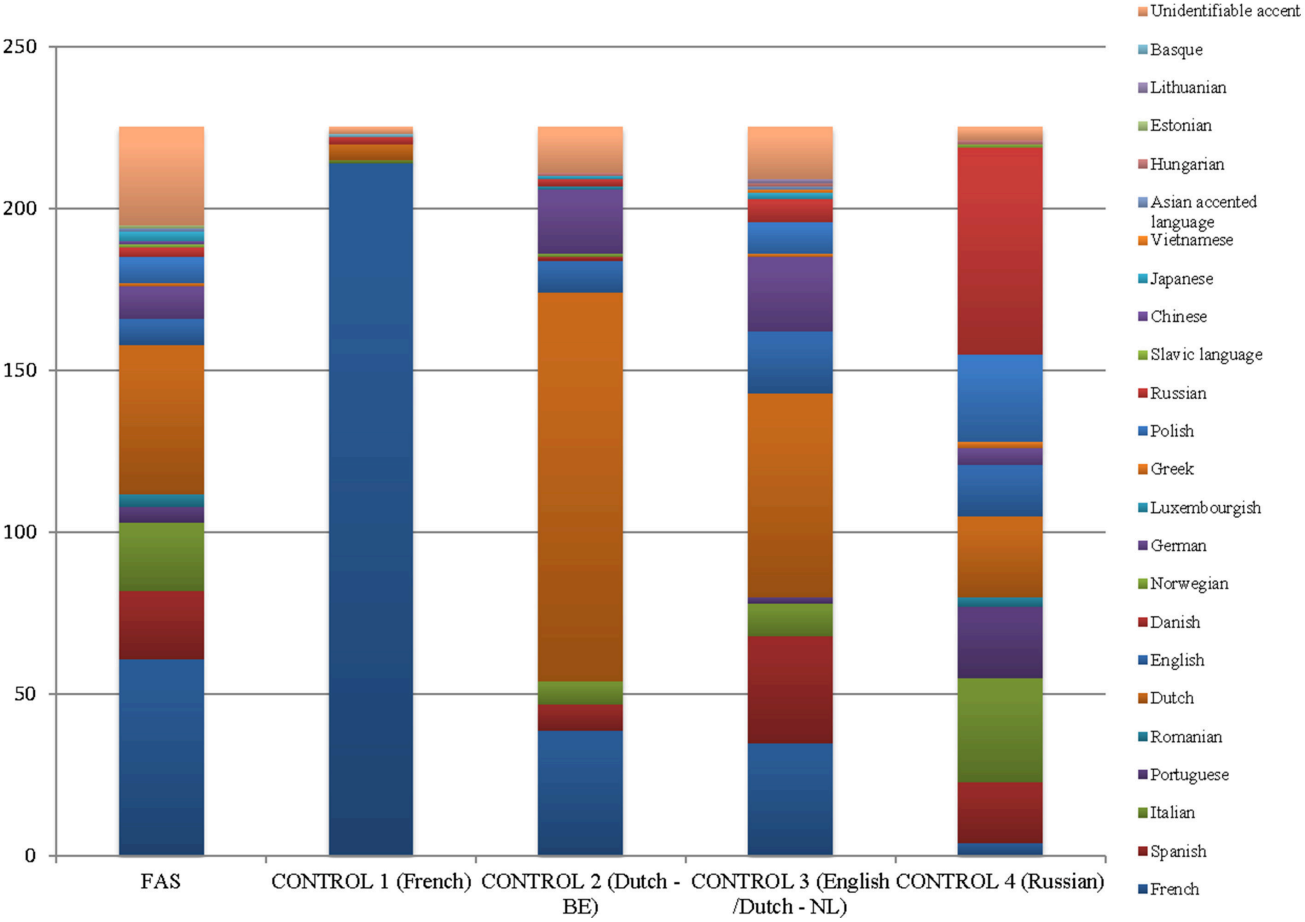
**Graphical overview of the stratification of the different mother tongues associated with the different speakers**.

It appeared that only 50% of the raters (*n* = 15/30) had indicated the mother tongue of each speaker for each stimulus. Nine raters were female, and six were male (age range: 16–23 years; mean age: 19 years). Figure 2 shows the different accents associated with the different speakers. Exact numbers can be found in Table 6.

**Table 6:** **Overview of the mother tongues (rows) associated with each of the speakers (columns)**.

	**FAS**	**French**	**Dutch**	**English/Dutch**	**Russian**
French	61	214	39	35	4
Spanish	21	0	8	33	19
Italian	21	1	7	10	32
Portuguese	5	0	0	2	22
Romanian	4	0	0	0	3
Dutch	46	5	120	63	25
English	8	0	10	19	16
Danish	0	0	1	0	0
Norwegian	0	0	1	0	0
German	10	0	20	23	5
Luxembourgish	0	0	1	0	0
Greek	1	0	0	1	2
Polish	8	0	0	10	27
Russian	3	2	2	7	64
Slavic	1	0	0	0	1
Chinese	1	0	0	0	0
Japanese	3	0	1	2	0
Vietnamese	0	0	0	1	0
Asian-sounding accent	1	0	0	1	0
Hungarian	0	0	1	1	1
Estonian	1	0	0	0	0
Lithuanian	0	0	0	1	0
Turkish	0	0	0	0	0
Basque	0	1	0	0	0
Unidentifiable	30	2	14	16	4
TOTAL	225	225	225	225	225

Languages pertaining to the same language family have been grouped together.

In general, the FAS patient was less often identified as “French” (*n* = 61/225; 27.1%) than as a speaker of other languages (72.9%). However, the other languages attributed to the FAS patient were most often Romance languages (Spanish: *n* = 21; Italian: *n* = 21; Portuguese: *n* = 5; Romanian: *n* = 4; *n* = 112/225; 49.8%). Still, she was identified as Dutch in 21.3% of the stimuli (*n* = 48/225), and as German in 4% of stimuli (*n* = 10/225). The FAS patient was less often identified as “French” than the native French speaker (*n* = 214/225; 95.1%), which corroborates the findings for the first part of the study. Hence, there seemed to be a clear difference in the perception of the FAS patient and the non-impaired French control speaker. The Dutch (Be) speaker was associated with “Dutch” in 53.3% (*n* = 120/225) of the stimuli, whereas for the English/Dutch(Nl) speaker this was 28% of the stimuli (*n* = 63/225). In 8% of the stimuli she was associated with “English” (*n* = 19/225). The Russian speaker was correctly associated with her native language in 28.4% (*n* = 64/225) of the stimuli.

Interestingly, the accent stratification was most diverse for the FAS patient (16 different mother tongues were associated with her stimuli). For the other speakers, the number of attributed accents was: English/Dutch(Nl): 15; Russian: 13; Dutch (Be): 12; and French: 5. Equally interesting to note is that the accent of the FAS patient could not be identified in 30 items: this is considerably more often than for the other control speakers: French: 2; Dutch (Be): 14; English/Dutch (Nl): 16; Russian: 1.

## Discussion

This article discusses the case of a patient who developed FAS in the absence of demonstrable damage to the central nervous system. No structural damage was visible on repeat CT and MRI of the brain. Repeat neurological and neurophysiological examinations were normal. An in-depth psychodiagnostic work-up was carried out (a) to confirm the existence of psychological issues and (b) to identify a possible psychiatric disorder. Unfortunately, testing did not reveal a clearly delineated disorder on either axis I or II of the DSM-IV-TR (APA, 2000). Test results were, however, indicative of a highly dependent, hysterical and borderline personality. Although psychological problems were considered persistent and chronic, there were several elements in the clinical interviews that could corroborate the hypothesis of a psychogenic origin of the accent change.

First, the accent diminished whenever there was a psychological breakthrough during the clinical interview (Avbersek and Sisodiya, 2010; see also: Keulen et al., 2016,^2^). More specifically these episodes occurred when the patient talked about her relational problems, issues at her former workplace and the fact that she no longer had a job at the moment the interview took place. Interestingly, the *negative* impact of emotional disequilibrium, feelings of stress and/or anxiety on the recovery process has previously been established for neurological speech and language disorders (see also: Cahana-Amitay and Albert, 2015). In contrast, current patient seemed to *benefit* from these emotional triggers.

Second, there was a correspondence between the culmination of disputes with her line manager, which ultimately led her dismissal, and the onset of the accent: both occurred approximately 6 months after the accident.

Third—and related to prior argument—the increased emotional lability and hysteric symptoms may have been reinforced by the adverse life events that had marked her life in rapid succession: the car accident, the accent shift, the dismissal, and the relational problems. According to Avison and Turner (1988) the relationship between adverse life events and psychological distress is often underestimated. According to Charles et al. (2013) even naturally occurring daily stressors or minor affective experiences can have a far-reaching impact on mental health (p. 739). It is important to note that at the time we saw the patient, she had been unemployed for about a year and a half and had marital problems.

The patient had repeatedly complained about (sustained) attentional and amnestic problems, as well as slow cognitive processing. These complaints were confirmed by neuropsychological test results: the patient demonstrated impaired processing on the cognitive tasks appealing to working memory, attention, and executive function. These complaints have been noted regularly in psychogenic FAS patients (Poulin et al., 2007; Cottingham and Boone, 2010; Jones et al., 2011; case Roy et al., 2012) and have more generally been associated with somatic disorders (Niemi et al., 2002; Trivedi, 2006; Demir et al., 2013). However, studies claiming such an association have been the subject of scientific scrutiny, because hardly anyone administered symptom validity tests to their participants prior to inclusion. Delis and Wetter (2007) suggest that patients with psychogenic disorders may exaggerate cognitive deficits, due to external (medico-legal reasons, treatment), internal/interpersonal incentives (in order to sustain a dependent relationship with specialist or other) or even for unspecified reasons (“not otherwise specified”). The current patient completed symptom validity tests, which turned out negative for malingering and feigning. Moreover, neurocognitive testing was carried out on two occasions (2 years apart, both post-morbid). This is crucial, as significant underperformance or inconsistencies in cognitive test scores or profiles across repeated evaluation would be considered indicative of a feigned cognitive deficit.

In the case of our patient the profile seems mostly consistent with a post-concussion cognitive syndrome after a minor head trauma. The objectively attested cognitive deficits and the negligence of the cognitive complaints after prior examinations might also have contributed to the development of the FAS.

On a linguistic level, the patient's speech was characterized by the realization of the uvular R with a marked, atypical trill and occasionally, she deleted phonemes. Furthermore, the patient spoke at a very slow speech rate and had a speech rhythm that was qualified as stress-timed, whilst French is a syllable-timed language (Grabe and Low, 2002). The segmental and suprasegmental characteristics noted for this patient do not seem to be restricted to a psychogenic population: all have been attested for neurogenic FAS patients as well. However, the isolated, morphological deficits, which irregularly affected articles, and the occasional pronunciation deficits affecting liaisons and elisions (phenomena typifying French) seemed incredible. The grammatical deficit is very different and less substantial than the agrammatism and paragrammatism encountered in aphasics, for instance. Some degree of conscious or subconscious manipulation cannot be ruled out. Incredible grammatical disorders of the like have previously been reported in other psychogenic cases (e.g., Van Borsel et al., 2005; Cottingham and Boone, 2010).

Some speech characteristics might have been consistent with the impression of a Dutch or German accent. However, results of the listening experiment suggest that the patient was perceptually situated midway between a true non-native speaker of French and a native speaker of French. This finding is in line with what has been found in the experiments of Di Dio et al. (2006), Kanjee et al. (2010) and Verhoeven et al. (2013). However, the methodology in Verhoeven et al. (2013) and Kanjee et al. (2010) differed from the current one in the sense that they did not select words and sentences in pseudo-random order, but provided raters with spontaneous speech samples (Verhoeven et al., 2013) or elicited (read) sentences (Kanjee et al., 2010). For Di Dio et al. (2006) it is not clear what type of stimuli was used. The methodology in the present experiment was more comparable to the approach of Dankovičová and Hunt (2011), who used single words and phrases. As far the identification of the linguistic background of the speakers is concerned, it was found that the FAS patient was associated with French in only 27.1% of the stimuli. The French-speaking control subject on the other hand was almost always recognized as French (95.1%). The patient also demonstrated the most diverse association patterns regarding her native language. The “uncertainty” expressed in the first part of the experiment (*M* = 4) compares well with the second part of the study: the patient was associated with 16 different possible native languages, and for 13% of the items, the mother tongue could not be identified. Furthermore, the hypothesis that the patient was perceived as Dutch or German was not entirely confirmed, as most listeners still perceived her as being a native speaker of a Romance language (for 49.8% of the stimuli, including French, Italian, Spanish, Portuguese, and Romanian; in comparison: Germanic languages, including English, Dutch and German: 28.4% of the stimuli).

Remarkably, the patient did not seem bothered by the accent change at all. Nevertheless, there were clear problems at the cognitive-behavioral and psychological level (mentioned above). Moreover, she was not keen to be treated for the condition. Rather, she wanted to show off with it. She did not seem to be overtly concerned about her symptoms. This is unlike what is mostly seen in neurogenic patients, who are emotionally and psychologically affected by FAS (Miller et al., 2011). In fact, to the best of our knowledge, there are only two other reports (Laures-Gore et al., 2006; Tailby et al., 2013) in which it was mentioned that patients were almost completely indifferent to the negative implications. These cases were classified as “mixed FAS” by Verhoeven and Mariën (2010): these patients further optimize their accent and often start to use words of the language, which is suggested by their accent in order to create a more authentic personality. The use of foreign-sounding words or a more formal language variant has also been noted for psychogenic FAS patients (Reeves and Norton, 2001; Poulin et al., 2007; see also: Reeves et al., 2007, case 3; Polak et al., 2013). The processes which invoke this kind of change in language use, still remain to be clarified. For neurogenic cases, some positive associations have also been noted. According to some patients, living with FAS opened new horizons. However, in the longer term, the negative perceptions from others, the hybrid identity, a loss of sense of belonging, a breakdown of relationships, and the incapacity of medical staff to explain the change all lead to frustrations (Miller et al., 2011).

Patient coping strategies, psycho-emotional and -social implications have generally been underreported in the literature about both psychogenic and neurogenic FAS (for neurogenic patients: Munson, 2005; Miller et al., 2006, 2011; Moreno-Torres et al., 2013). Future research should identify and study the effects of this syndrome at the personal and inter-personal level to allow for a full rehabilitation of both speech profile and psychological well-being.

## Concluding remarks

Only a handful of putative psychogenic FAS cases have been described in the literature and many researchers have been hesitant to conclude to an underlying psychogenic etiology. Although it is hard to provide evidence for a direct causal link between the psychological factor in play and FAS, ample evidence exists that the FAS symptoms (and their course) in this patient are of a psychogenic nature: (1) clear absence of (visible) neurological damage or clinical evidence for a neurological disorder, in conjunction with (2) the presence of psychological and psychiatric factors, (3) the timing of the onset of the accent change, (4) the atypical and fluctuating symptom course, (5) irregular and incredible morphological mistakes occurring in a short sample of spontaneous speech, and the fact that (6) the patient was unconcerned by the change of accent. As most of the psychogenic FAS cases were published in the last decades, reports of cognitive-behavioral deficits such as the ones displayed by current patient are becoming increasingly important with a view to the development of the proper therapeutic approaches for this psychogenic FAS population.

## Author contributions

Acquisition of data: SK, LDP, and PM. Analysis and interpretation of data: SK, JV, RJ, LDP, RB, and PM. Drafting the manuscript: SK and PM. Critical manuscript revision: all authors. Critical revision of reviewed manuscript: SK, JV, and PM. Final manuscript approval: SK and PM on behalf of all authors.

### Conflict of interest statement

The authors declare that the research was conducted in the absence of any commercial or financial relationships that could be construed as a potential conflict of interest.

## Footnotes

^1^In English, the term elision is sometimes used as a synonym for deletion (e.g., Miller et al., 2006). For current article, we make a distinction between a “deletion” and an “elision” (French: “*élision”*), which is the “the suppression of a word-final vowel preceding a word starting with a vowel” (in spoken French this can refer to actual vowels, or the latent word-initial “*h*” preceding a vowel—with a few exceptions; Schane and Filloux, 1967, p. 37, *our translation*).

^2^In this recently published article, another case of psychogenic FAS is presented. The patient suddenly lost her accent during a temper tantrum.
